# Home Sweet Home: *Plasmodium vivax*-Infected Reticulocytes—The Younger the Better?

**DOI:** 10.3389/fcimb.2021.675156

**Published:** 2021-05-13

**Authors:** Richard Thomson-Luque, José M. Bautista

**Affiliations:** ^1^ Center of Infectious Diseases, Parasitology, Heidelberg University Hospital, Heidelberg, Germany; ^2^ Department of Biochemistry and Molecular Biology and Research Institute Hospital 12 de Octubre (Imas12), Universidad Complutense de Madrid, Madrid, Spain

**Keywords:** malaria, reticulocytes, host cell, *Plasmodium vivax*, fitness

## Abstract

After a century of constant failure to produce an *in vitro* culture of the most widespread human malaria parasite *Plasmodium vivax*, recent advances have highlighted the difficulties to provide this parasite with a healthy host cell to invade, develop, and multiply under *in vitro* conditions. The actual level of understanding of the heterogeneous populations of cells—framed under the name ‘reticulocytes’—and, importantly, their adequate *in vitro* progression from very immature reticulocytes to normocytes (mature erythrocytes) is far from complete. The volatility of its individual stability may suggest the reticulocyte as a delusory cell, particularly to be used for stable culture purposes. Yet, the recent relevance gained by a specific subset of highly immature reticulocytes has brought some hope. Very immature reticulocytes are characterized by a peculiar membrane harboring a plethora of molecules potentially involved in *P. vivax* invasion and by an intracellular complexity dynamically changing upon its quick maturation into normocytes. We analyze the potentialities offered by this youngest reticulocyte subsets as an ideal *in vitro* host cell for *P. vivax*.

## Introduction

*Plasmodium vivax* (*P. vivax)* is the reigning malaria-causing parasite outside the African continent (Gallup and Sachs, 2001). The strong morbidity burden carried by populations living in areas endemic for this understudied parasite rebounds in the chronic impoverishment and underdevelopment of these communities (WHO, 2016). For a long time, *P. vivax* has been considered as the causing agent of the historically—yet inaccurately—termed “*benign malaria*”. This inappropriate stigma has disregarded *P. vivax* at the end of the row in terms of malaria research priorities (Mueller et al., 2009). Happily, in the last decade, there have been remarkable efforts to promote *P. vivax* research for its capacity to remain dormant in the liver in the form of hyponozites for long periods of time and then relapse (Krotoski, 1985). Insights into intriguing biological features of *P. vivax*, such as the real contribution of the hematopoietic niches in bone marrow and spleen (with extramedullary erythropoiesis potentially occurring under adverse conditions, including malaria) as homes for a significant proportion of parasite biomass, are also on the increase (Silva-Filho et al., 2020). Advancment in this field is very much warranted due to the potential of the reticulocyte’s intracellular environment to trigger the sexual commitment of *P. vivax* (Obaldia et al., 2018) and subsequent importance for transmission and eradication efforts. For this and its very early sexual commitment in transmissible-stage gametocytes (Adapa et al., 2019), it seems plausible that *P. vivax* may become the last human *Plasmodium* parasite standing before the goal of malaria eradication is achieved (Tanner et al., 2015).

A much-needed tool is still missing to boost research into *P. vivax*’s intrinsic biological and pathophysiological singularities to the level that we have today for *Plasmodium falciparum* (*P. falciparum*), for which an *in vitro* culture system for blood stages has existed for more than 40 years (Trager and Jensen, 1976). This availability has allowed us to genetically unravel *P. falciparum*’s genes functionalities through already in place forward and reverse genetic approaches (Kirchner et al., 2016; Zhang et al., 2018), proteomics (Bautista et al., 2014), or immunomics (Doolan, 2011), which can ultimately lead to more rational development of new antimalarial drugs and promotion of vaccines candidates (Bourgard et al., 2018).

Several breakthroughs in the cultivation of *P. knowlesi* (Moon et al., 2013; Grüring et al., 2014) and *P. cynomolgi* (Chua et al., 2019) have been achieved in the last decade. As for *P. vivax*, we are still missing a reliable method for its *in vitro* culture; the major impediment has been our inability to efficiently handle it under *in vitro* culture conditions its sole target cell for asexual blood-stage replication: the reticulocyte (Thomson-Luque et al., 2019). Improved methods for reticulocyte enrichment from different sources have been provided (Vettore et al., 1980; Brun et al., 1990; Kumar et al., 2015; Shaw-Saliba et al., 2016), as well as the production of reticulocytes through better optimized hematopoietic stem cell (HSC) cultures (Giarratana et al., 2005, Noulin et al., 2013; Scully et al., 2019) and immortalized lines (Satchwell et al., 2019; Heshusius et al., 2019; Trakarnsanga et al., 2020). The lack of a more efficient enucleation (Menon and Ghaffari, 2021) can be overcome by genetic complementation (Scully et al., 2019). Humanized mouse models, such as the human liver-chimeric FRG KO huHep to recapitulate the liver, and blood-stage cycles of *P. vivax* (Mikolajczak et al., 2015; Schäfer et al., 2020) are readily available, although at a high cost and low efficiency in terms of blood-stage breakthrough; the liver stage *in vitro* systems (Roth et al., 2018) are currently being optimized to unravel the mechanism of hypnozoite production, though again at a high cost. Furthermore, non-human primate monkey models, such as Aotus, Saimiri, and Rhesus, are also a possibility to study this parasite *in vivo* (Shaw-Saliba et al., 2016; Pasini and Kocken, 2021), although rising ethics concerns makes this model only available to certain facilities. Thus, all expectations are put on the development of affordable *in vitro* cultures, and, for this, a substantial leap in healthily handling *in vitro* reticulocytes to offer *P. vivax* the right host cell capable of providing specific receptors and an intracellular niche for the parasite to mature and replicate, is still needed. This is the way forward.

## The Reticulocyte: not a Specific Cell Type but a Continuum in Maturation Difficult to Reproduce *In Vitro*

**Figure 1:**
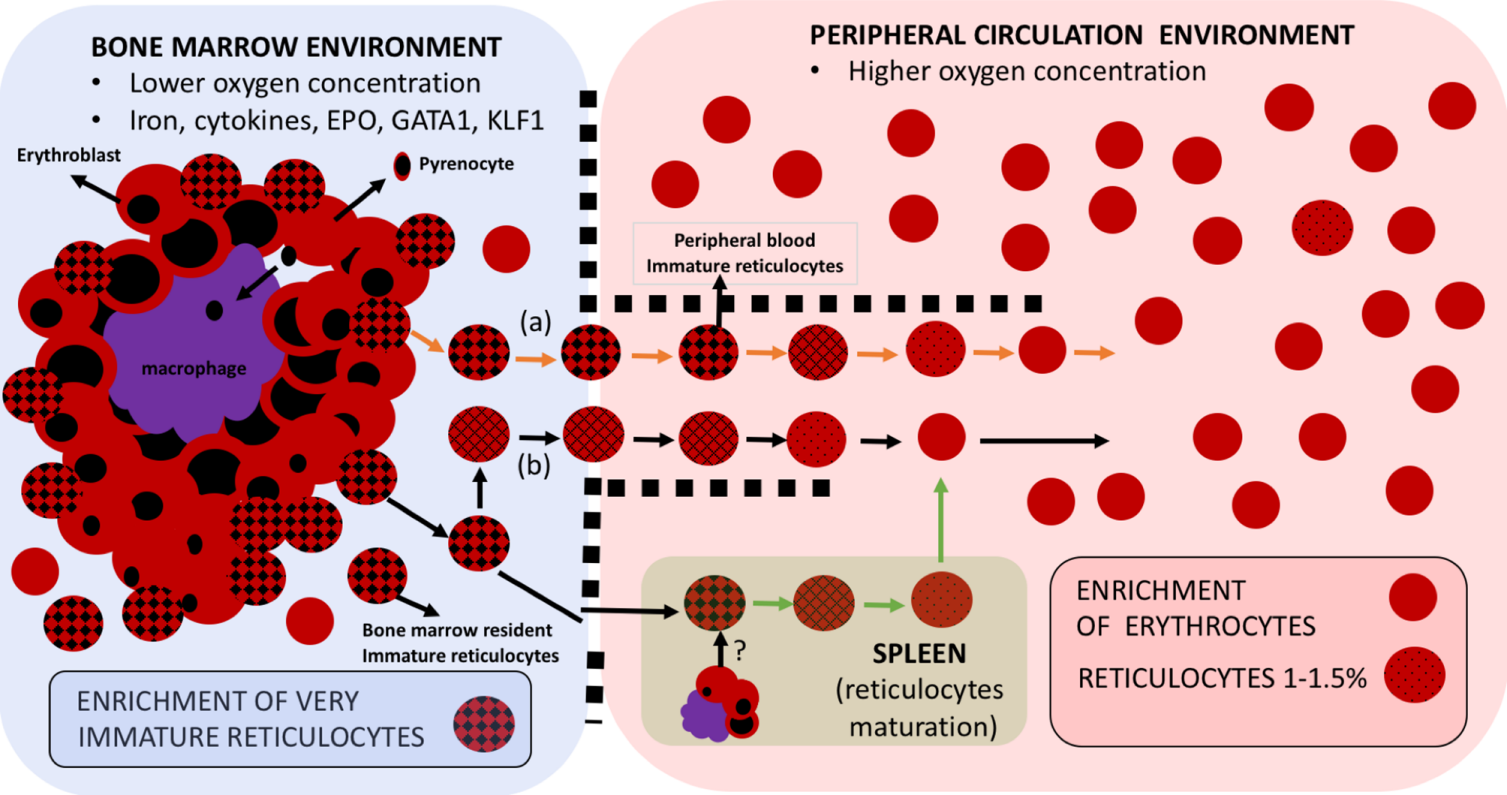
Erythroid cell maturation occurs in different compartments. Reticulocytes emerge in the hypoxic bone marrow compartment upon their predecessor cell in the erythroid lineage, the orthochromatic erythroblasts, expelling its nucleus, in the form of a pyrenocyte, which will be thereafter engulfed by the key scaffold cell of the erythroblastic islands, the central macrophage. These macrophages also play an important role in secreting cytokines that will contribute to the maturation of the whole erythroid lineage from erythroid committed stem cells to CFU/BFU and all the way to reticulocytes. Some recently-enucleated reticulocytes may leave the bone marrow to start maturation in peripheral blood **(A)**, But mostly they start maturing within the bone marrow compartment **(B)** to ultimately progress to fully mature RBCs in the peripheral blood compartment with higher oxygen concentration. Immature reticulocytes, sometimes referred to as CD71^high^ reticulocytes are thus preferentially enriched in the bone marrow but can also be found in peripheral circulation. The spleen represents a hematopoietic organ with potential for erythropoiesis under stress circumstances and where reticulocyte maturation is postulated to happen.

The persisting and adverse scenario of the lack of an *in vitro* culture system for *P. vivax* is indicative of the lack of understanding of the reticulocyte biology *in vitro*. Far from a homogeneous cluster of erythroid cells, reticulocytes are a population in constant phenotypical change. The most immature reticulocytes formed in the bone marrow’s erythroblastic islands continuously develop, both internally in its cytoplasm as well as in its external surface membrane, to finally become, while in circulation, fully mature red blood cells (RBC) called normocytes (**Figure 1**). Different approaches aimed at characterizing these dynamic reticulocytes have (imperfectly) attempted to establish a classification to by focusing on different features of reticulocytes. The earliest, the Heilmeyer staging I-V focused on their microscopical appearance after New Methylene Blue (NMB) staining (Heilmeyer and Westha üser, 1932). Later, they were classified as R1 vs R2 reticulocytes groups based on the shape and movement of reticulocytes in live cell cytology studies (Mel et al., 1977). More recently, the amount of transferrin receptor 1 (TfR1 or CD71) expression in the membrane of reticulocytes (Brun et al., 1990; Kono et al., 2009) has become the current trending classification. Remarkably, all three phenotypic viewpoints must be acknowledged and taken into consideration when attempting to unravel keys to efficiently sustain healthy reticulocyte populations in an *in vitro* culture system.

Maintaining, under *in vitro* conditions, the correct fitness of these heterogeneous cells at all steps throughout their developmental continuum is thus paramount to advance in establishing a *P. vivax in vitro* culture; but this turns out to not be an easy task. Not enough importance has been given to the fact that the widely accepted 2–3 days necessary for a correct *in vivo* maturation of reticulocytes (Seip, 1953) takes place in between two compartments with the divergent environment. For instance, from the low oxygen concentration niche at the bone marrow, reticulocytes depart to a highly oxygenated milieu into the peripheral blood where they finally reach and transform into normocytes (Tugba et al., 2010). This maturation when pursued *in vitro* is very much inhibited (Malleret et al., 2013). Size and morphological discrepancies are also observed between *ex vivo* (Malleret et al., 2013) and *in vitro* maturation (Griffiths et al., 2012). Further exploration into adjusting the *in vitro* development of reticulocytes under hypoxic culture conditions, at least partially during the first steps of maturation, is desirable to satisfactorily provide reticulocytes with an ideal environment from the very first moments of its maturation to finally obtain the healthiest cell that could support efficient parasite invasion and correct development.

## CD71^high^ Reticulocytes: a Promising Reticulocyte Subtype to Identify Missing Receptors/Co-Receptors

Our limited knowledge of reticulocyte molecular mediators of invasion by *P. vivax* contrasts with the vast repertoire reported for *P. falciparum* invasion (Beeson et al., 2016). The clear role of the Duffy antigen receptor for chemokines (DARC/CD234) in red blood cells (RBCs) as a receptor for the DBL domain in Region II of *P. vivax* (DBPII) is clearly established (Adams et al., 1992). However, DARC is present in both reticulocytes and normocytes and its protein expression does not change during reticulocyte maturation. The use of monoclonal antibodies to specific epitopes within DARC has pointed at an increased DARC-DBP binding site accessibility in reticulocytes and, importantly, in very immature reticulocytes (Ovchynnikova et al., 2017). Yet, the strict tropism of *P. vivax* for reticulocytes (Kitchen, 1939; Mons, 1990) cannot be totally explained by this well-described molecular interaction. Several receptors/co-receptors-ligand interactions have been envisioned to unravel *P. vivax* invasion pathway/s (Ménard et al., 2013; Ntumngia et al., 2016). Recently, CD71 present in younger reticulocytes (CD71+ reticulocytes) has been promoted as the receptor for the *P. vivax* ligand reticulocyte-binding protein 2b (RBP2b), shedding stronger insight into the strict reticulocyte attraction by *P. vivax* (Gruszczyk et al., 2018a and Gruszczyk et al., 2018b). The suggested dependency on CD71 for invasion has furthermore re-fueled the idea that a great proportion of *P. vivax* biomass resides in hematopoietic organs, such as the bone marrow (Baird, 2013) (and potentially the spleen, contributing to the final steps of reticulocyte maturation) (Rhodes et al., 2016; Toda et al., 2020). These are environments full of the younger CD71+ reticulocytes and, particularly, the homes of a subset of reticulocytes whose surfaces are extremely enriched in CD71: the CD71^high^ reticulocytes. These most immature reticulocytes are nowadays considered as the key reticulocyte subset to unlock the way for obtaining an *in vitro* culture system for *P. vivax*.

Although an attractive proposal, there is currently no definitive *in vivo* evidence on a clear tropism of *P. vivax* to invade the youngest reticulocytes when infecting humans. Since the first report on the presence of *P. vivax* in bone marrow aspirates in humans (Marchiafava and Bignami, 1984), the following findings of this parasite in hematopoietic organs till present have been merely incidental (Lacerda et al., 2008; Baro et al., 2017). Histological analysis in a nonhuman primate model has just reflected a moderate enrichment of asexual stages in the parenchyma (Obaldia et al., 2018). These data cannot firmly support that the subset of reticulocytes being invaded *in vivo* are only bone marrow-residing CD71^high^. Yet, in an *in vitro* experimental setting, an immature CD71^high^ reticulocyte subpopulation as preferentially chosen for invasion by *P. vivax* (Malleret et al., 2015). This has likewise been demonstrated for the reticulocyte-prone rodent malaria parasite *Plasmodium yoelii* 17X *NL* (Martín-Jaular et al., 2013). This finding has promoted investigating molecules present in the membrane of CD71^high^ that may act as yet-unreported receptors involved in *P. vivax* invasion.

Phenotypical characterization of reticulocytes in cord blood samples has tightly measured the abundance of many different RBC surface markers among different subsets showing that reticulocyte maturation is concomitant with decreasing CD71 expression (Malleret et al., 2013; Wilson et al., 2016; Chu et al., 2018). This has been later expanded to peripheral blood and bone marrow samples to study a broader set of markers (Thomson-Luque et al., 2018), and corroborating that, although at low levels compared to bone marrow, reticulocytes with very high CD71 loads can be released very rapidly to peripheral blood (even during the first 30 minutes after detaching from erythroblastic islands when assuming a linear age distribution) (Ovchynnikova et al., 2017) (**Figure 1**). Therefore, if *P. vivax* invasion is specifically restricted to reticulocytes with the highest CD71 expression, replication could also occur in peripheral circulation. Other surface molecules such as CD49d (α4β1integrin), which is drastically lost at the very early hours of reticulocyte maturation, CD44 (Indian blood group), and CD98 are enriched in the most immature CD71^high^ reticulocytes (Griffiths et al., 2012). Consequently, wide cell-surface screenings are particularly relevant if some of these molecules are ever promoted as a potential receptor to explore.

## Young Reticulocytes: the Keystone for *P. vivax* Infection of the Duffy Negative African Population?

*P. vivax* infections have long been considered to be inexistent in the African continent (Miller et al., 1976). The clear contrast in the geographic distribution among *P. vivax* and the deadliest species in Africa *P. falciparum* has been historically based on the imperative of *P. vivax* to invade human populations positive for DARC, which presence is very limited in Africa (Howes et al., 2011). Yet, initial reports have put on the table the potential ability of *P. vivax* to cause disease all the way from East (Bray, 1958; Ryan et al., 2006; Ménard et al., 2010; Woldearegai et al., 2013; Lover, 2014) to Central (Culleton et al., 2009; Wurtz et al., 2011) and West Africa (Fru-Cho et al., 2014; Niangaly et al., 2017; and extensively reviewed in Popovici et al., 2020). The question of how *P. vivax* merozoites invade reticulocytes from African populations not carrying DARC (Gunalan et al., 2018) can be answered through *in vitro* dissections of invasion ligand/host receptor interactions.

Studies of various culture conditions in red cells have revealed switching mechanisms in mature RBCs invasion by *P. falciparum* (Dolan et al., 1990). Recently, by blocking reticulocytes’ DARC and TfR1 receptors in short-term cultures of *P. vivax* isolates, a significant variation in receptor usage was observed, suggesting that *P. vivax* may use alternative invasion pathways (Kanjee et al., 2020). Immature reticulocytes clearly represent the right cell to search for these alternative pathways’ mediators.

Moreover, the leaky expression of DARC on Duffy negative (Duffy^-^) RBCs has been previously suggested (Gunalan et al., 2020) as a potential explanation for *P. vivax* invasion into Duffy^-^reticulocytes. Some DARC^-^ individuals may not be fully Duffy-null, as residual RNA transcription may still happen marginally (Popovici et al., 2020). This phenomenon has recently been described in bone marrow-derived DARC^-^ RBC progenitors (Dechavanne et al., 2018). The possibility that some very immature DARC^-^ reticulocytes in the bone marrow, but also in peripheral blood (Thomson-Luque et al., 2018), harbor marginal expression of DARC tempts us to speculate that they may be the explanation behind the possibility of transmission between Duffy^-^ individuals infected with *P. vivax.* This is further supported if the molecules characteristic of reticulocyte immaturity that they carry have indeed a role as alternative receptors/co-receptors. However, aside from receptor molecules, other players constitutive of the immature reticulocyte membrane may need to be looked into and taken care of under *in vitro* conditions.

## A Constant Remodeling of the Reticulocyte Membrane *in Vitro* is Needed

**Figure 2:**
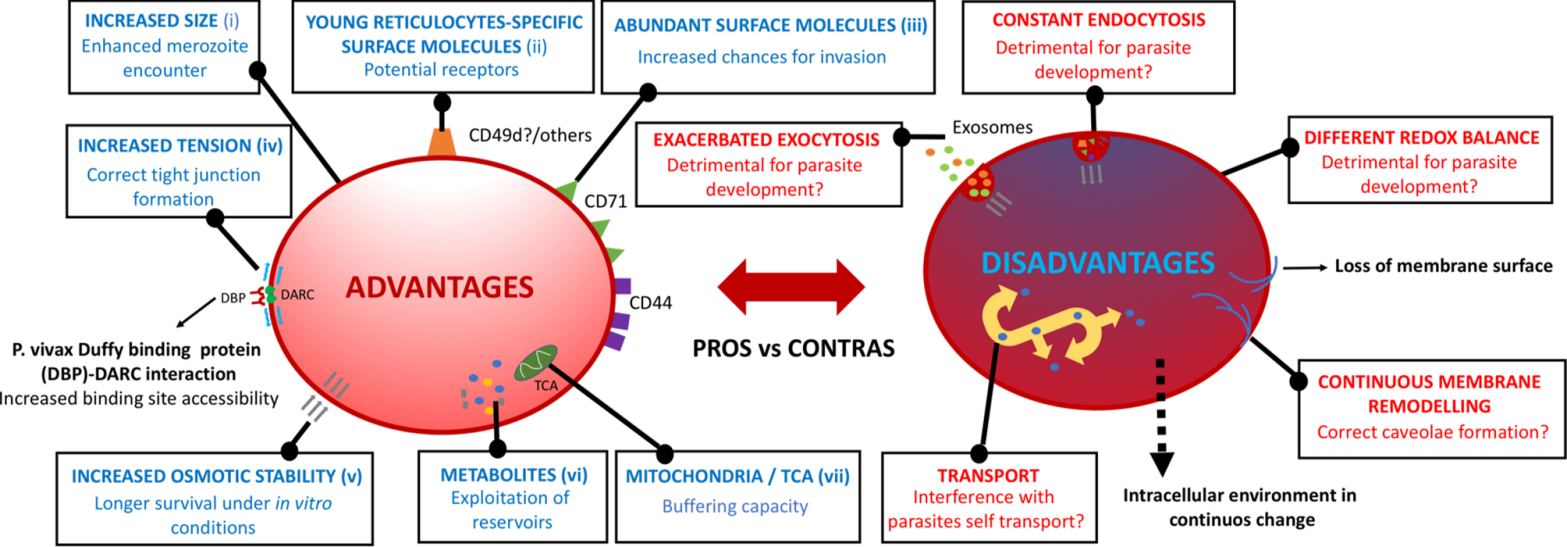
Potential advantages and disadvantages of immature reticulocytes. Surface and intracellular phenotypic features at the very initial steps of very immature reticulocyte maturation that may confer/impede the *P. vivax* parasite’s subsequent physiological progression throughout their intra-reticulocytic developmental cycle. (i) Griffiths et al., 2012, (ii) Thomson-Luque et al., 2018, (iii) Malleret et al., 2013, (iv) Kariuki et al., 2020, (v) Clark et al., 2021, (vi) Srivastava et al., 2015, (vii) Starkov, 2008.

A healthy reticulocyte membrane is not just needed as a cytoskeletal platform [composed of networking molecules spectrin, actin, tubulin, ankyrin, adducin, tropomyosin, and tropomodulin, linking the major structural elements protein 4.1 and band 3 (Liu et al., 2010)] to anchor receptor molecules. Such a structured membrane is also requested to support the biophysical requirements for invasion, establishing the correct tension for the DBP-DARC tight junction and, potentially, of unidentified receptor/ligand interactions, to efficiently interact (Kariuki et al., 2020). DARC expression dependency on the junctional complex with protein 4.1 supports this fact, as protein 4.1 deficiency reduces the expression of DARC (Salomao et al., 2008; Azouzi et al., 2015). The gradual remodeling of the reticulocyte membrane’s nanostructure (Li et al., 2018), involving the loss of up to one-third of its surface area (Griffiths et al., 2012), has been widely studied with the use of a variety of approaches, such as SEM, TEM, micropipette aspiration, and atomic force microscopy (Malleret et al., 2013; Malleret et al., 2015). Yet again, very little is known of the correct cytoskeleton maturation of reticulocytes *in vitro*, with membrane dismembering being common under standard *P. vivax* culture settings (Thomson-Luque et al., 2017). A decrease in osmotic stability has been shown to be a major cause for the loss of structural integrity of reticulocytes undergoing *in vitro* maturation in HSC cultures (Clark et al., 2021). This suggests that immature reticulocytes are more osmotically stable, pointing to an advantage for *P. vivax* to develop when invading these cell subtypes (**Figure 2**). Yet, a relevant uncertainty remains as to whether reticulocyte’s membrane maturation is modified as a consequence of the *P. vivax* infection itself.

A reported acceleration in the maturation of cord blood-derived reticulocytes triggered by *P. vivax* right after merozoite invasion *in vitro* has described a fast decline of CD71 from the surface of *P. vivax-*infected CD71+ reticulocytes (Malleret et al., 2015). This is accompanied by rapid loss of the inner reticulae (a conglomerate of polyribosomes, RNA, endothelium reticulum, and sometimes mitochondria) 3 hours post-invasion. These events are found to go hand to hand with a concomitant shift in membrane nanostructure components, from clathrin pit-enriched towards caveolae-enriched infected reticulocyte membrane, as the parasite matures in an *in vitro* environment. In the context of these findings we need to consider the following: (i) the accelerated disappearance of CD71 is not homogeneous, and some *P. vivax* late stages are inside some CD71+ reticulocytes; and (ii) the discrepancy with findings of *P. vivax* late stages inside NMB+ reticulocytes (Lim et al., 2016) as well as inside CD71+ reticulocytes (Clark et al., 2021) in patients’ field isolates. In the *Aotus lemurinus lemurinus* monkey malaria model (Shaw-Saliba et al., 2016), co-staining of *in vivo* samples containing *P. vivax* infected reticulocytes with both Giemsa and supravital New Methylene blue revealed that in *Aotus* monkeys *in vivo P. vivax* Sal-1 of different developmental stage can be found inside reticulocytes of different maturity (from Heilmeyer I to IV). In addition, the second generation of these parasites *in vitro* cultured for 20 hours right after bleeding an infected monkey were also found inside Heilmeyer II to III reticulocytes [a subtype described as part of a CD71+ subpopulation of reticulocytes sorted by flow cytometry (Malleret et al., 2013)]. Yet, different *P. vivax* strains may vary largely in their reticulocyte preference (Lim et al., 2016) and infection variability regarding DARC polymorphisms (Fyb and Fya) should not be looked aside. Whether the fast remodeling is indeed triggered by the parasite or it is just an *in vitro* effect warrants further exploration; and furthermore, to answer as to what extent we need to experimentally pursue these fast dynamic changes in order to achieve the fittest *in vitro* culture possible for *P. vivax*.

## The Inner Intracellular Environment of Immature Reticulocytes and its Remodeling Pace

Fast events occurring in the membrane of reticulocytes upon maturation are highly intertwined with the massive inner remodeling. Understanding the intra-host cell environment and its corresponding changes upon the different maturity states of reticulocytes, and especially in the initial steps of CD71^high^ maturation, is crucial as these singular cells may provide metabolic reservoirs for *P. vivax* to take advantage upon developmental advancement (Srivastava et al., 2015) (**Figure 2**). In cord blood, for example, differing levels of amino acids, nucleotides, and sugars, among others, have been found in the different age-related subsets of reticulocytes, with decreasing concentrations as the reticulocyte matures (Darghouth et al., 2011; Malleret et al., 2013). Active metabolic pathways have been shown to remain in reticulocytes, whilst they tend to disappear in normocytes (Srivastava et al., 2017). Thus, the correct metabolite content and redox balance need to be mimicked in an *in vitro* setting as well, especially at the first steps of maturation, as slight differences may alter a wide range of fast processes occurring both in the membrane as well as in the cytoplasm.

Deleterious metabolic conditions in cultured reticulocytes may have a consequence in the clathrin pits-originated endocytosis mechanism for the sorting of disposable membrane proteins, such as CD71. Consequently, not leading to the ideal formation of multi-vesicular bodies due to the malfunction of the endosomal sorting complex required for transport, or even their correct fusion to the membrane, can have an effect on *P. vivax* invasion and development (Rieu et al., 2000). An altered redox regulation may also affect the ubiquitin-proteasome degradation pathway required for degrading cytosolic actin and tubulin (Liu et al., 2010). A functional tricarboxylic acid cycle (TCA cycle) is present in reticulocytes, consistent with the presence of residual mitochondria in the most immature subsets but lost thereafter (Srivastava et al., 2017) through a process of mitophagy (Lee et al., 2012). Whether the presence of mitochondria in the younger subset of reticulocytes is beneficial or detrimental for *P. vivax* to progress in the asexual cycle is also unknown. In favor of immature reticulocytes, mitochondrial by-products may be scavenged by the parasite for its own benefit, while the loss of mitochondrial observed in older reticulocytes could lead to the lack of enough buffering capacity against reactive oxygen species excess (Starkov, 2008) during parasite development, and therefore triggering host cell and parasite damage.

## Discussion: the Need for a More in-Depth Understanding of Immature Reticulocyte Fitness *In Vitro*

The advantage of using the youngest of the reticulocytes for facilitating *P. vivax in vitro* invasion seems sound, as its densely populated surface carries molecules potentially functioning as receptors. Due to the longer time to progress to normocytes, young reticulocytes can contribute to ameliorate the technical challenge of parasitemia dilution at every sub-culturing cycle resulting from the addition of new reticulocytes to the system characteristic of *P. vivax* cultures. The youngest reticulocytes can also provide the parasite with an extra supply of metabolites and a specific environment that is progressively lost as the reticulocytes mature to a low synthesizing cell. Some uncertainty may arise regarding the extent to which the hemoglobin provided by very young reticulocytes is enough for *P. vivax* as a source of amino acids. Yet, this would not seem a problem as *P. vivax* development inside even more immature nucleated erythroid cells, such as polychromatic erythroblasts, has been proven (Panichakul et al., 2007).

There have been a plethora of studies aiming at determining optimal culture media components to be used for *P. vivax in vitro* cultures to sustain not only parasite development but correct host cell survival (Roobsoong et al., 2015; Rangel et al., 2018; Thomson-Luque et al., 2019; Clark et al., 2021). A specific culture media recipe may as well need to be rationally designed and tested to specifically keep physiological reticulocyte remodeling (extensively reviewed in Thomson-Luque et al., 2019). Promoting this subpopulation of reticulocytes for its use for *in vitro* culture will require investing in an expensive and still inefficient large-scale isolation and storage, which is not available in every laboratory. More experimentation is clearly deserved on sustaining a parallel and healthy reticulocyte maturation of both its surface as well as internal components under *in vitro* conditions. Are both of these cytoplasmic and membrane maturations needed for *P. vivax* to develop inside? Is there a certain rate for a fine-tuned progression of *P. vivax* inside this delusory host cell? These are the key questions to be addressed; in order to gain more confidence in immature reticulocytes for achieving *P. vivax* culture *in vitro*, we must first discard that some of the described observations on the parasite’s biology are not just an artifact of non-viable reticulocytes in a non-optimized *in vitro* environment.

## Author Contributions

RT-L and JB wrote the manuscript. RT-L is funded by the European Union’s Horizon 2020 Research and Innovation Programme under Marie Skłodowska-Curie grant agreement DLV-839998. All authors contributed to the article and approved the submitted version.

## Funding

RT-L is funded by the European Union’s Horizon 2020 Research and Innovation Programme under Marie Skłodowska-Curie grant agreement DLV-839998. Part of this work has been funded by grant BIO2016-77430-R from the Ministerio de Economía y Competitividad (Spain).

## Conflict of Interest

The authors declare that the research was conducted in the absence of any commercial or financial relationships that could be construed as a potential conflict of interest.
